# Impact of drug classes and treatment availability on the rate of antiretroviral treatment change in the TREAT Asia HIV Observational Database (TAHOD)

**DOI:** 10.1186/1742-6405-4-18

**Published:** 2007-09-17

**Authors:** Preeyaporn Srasuebkul, Alexandra Calmy, Jialun Zhou, Nagalingeswaran Kumarasamy, Matthew Law, Poh Lian Lim, The TREAT Asia HIV Observational Database

**Affiliations:** 1 The National Centre in HIV Epidemiology and Clinical Research (NCHECR), University of New South Wales, Sydney, NSW, Australia; 2 St Vincent's Hospital, Sydney, Australia; 3 Division des Maladies infectieuses, unite VIH/SIDA, Hopital universitaire de Geneve, Switzerland; 4 YRG Centre for AIDS Research and Education, Chennai, India; 5 Tan Tock Seng Hospital, Singapore

## Abstract

**Background:**

It is critical to understand the pattern of antiretroviral treatment (ART) prescription in different regions of the world as ART procurement needs to be anticipated. We aimed at exploring rates and predictors of ART combination changes in clinical practice in Treat Asia HIV Observational Database (TAHOD).

**Methods:**

Rates of ART changes were examined in patients who started first line triple or more ART combination in TAHOD, and had at least one follow-up visit. Rates of ART changes were summarised per follow-up year, and factors associated with changes assessed using random-effect Poisson regression. The Kaplan-Meier method was used to determine durations of patients in their first, second and third regimen.

**Results:**

A total of 1846 patients initiated an ART combination with at least three drugs. Median follow up time for the first treatment was 3.2 years. The overall rate of ART change was 29 per 100-person-year.

In univariate analyses, rate of treatment change was significantly associated with exposure category, the country income category, the drug class combination, calendar year and the number of combinations. In multivariate analysis, compared to d4T/3TC/NVP, starting ART with another NNRTI-containing regimen, with PI only or with a triple NRTI regimen was associated with a higher risk of combination change (relative risk (RR) 1.6 (95% CI 1.64 – 1.96), p < 0.001, RR 3.39 (2.76 – 4.16) p < 0.001, RR 6.37 (4.51 – 9.00), p < 0.001). Being on a second or a third combination regimen was also associated with a decreased rate of ART change, compared with first ART combination (RR 0.82 (0.68 – 0.99), p = 0.035, RR 0.77 (0.61 – 0.97), p = 0.024). Sites with fewer than 12 drugs used had an increased rate of treatment changes (1.31 (1.13 – 1.51), p < 0.001). Injecting drug users, and other/unknown exposure was found to increase rate of treatment change (1.24 (1.00 – 1.54), p = 0.055). Percentages of patients who stopped treatment due to adverse events were 31, 27 and 32 in 1st, 2nd and 3rd treatment combinations, respectively.

**Conclusion:**

Our study suggests that drug availability impacts on ART prescription patterns. Our data, reflecting real clinic use in Asia, suggest that around half of all patients require second combination ART by 3 years after treatment initiation.

## Background

In South and South-East Asia the number of people living with HIV/AIDS in 2005 was 7,800,000, the second highest in the world after Sub-Saharan Africa [1]. Combination antiretroviral treatments have been widely available in Asia since 2003 [2].

The urgent need to provide antiretroviral therapy (ART) on a large scale resulted in a growing number of patients starting a simple, efficient, and standardized first line regimen. First line regimens usually include 2 nucleoside reverse transcriptase inhibitors (NRTI) and one non- nucleoside reverse transcriptase inhibitors (NNRTI), and this regimen can be co-formulated in a an easily administered fixed dose combination of d4T, 3TC and nevirapine [3]. Previous analyses have confirmed the efficiency of this approach [4-6].

Keeping the first line regimen as long as possible is considered essential. Adherence is of critical importance for long term durability because of the low genetic barrier to resistance for NNRTI-based regimens [7]. Experience from Western cohorts however shows that very few patients stay on a first regimen, with the median time of a first line regimen 1.6 years in a US cohort [8]. Previous analyses from the Australian HIV observational database (AHOD) showed that patients remain on their first treatment for a median 646 days (1.8 years) [9].

The range of drug options available in many Asian countries is not as wide as that available in developed countries. Moreover, the scale of epidemic implies that large numbers of patients need alternative first line or second line regimen. Reasons for switching are often related to treatment-related toxicity and adherence problems, and later in the course of the treatment, because of treatment failure [10,11]. Monitoring ART use in Asia is important: firstly, several countries in Asia have some of the highest patient loads in the world [12]. Secondly, Asian countries are very heterogeneous in terms of income access, pattern of the HIV epidemic, and treatment programs. In this paper, we explore the hypotheses that these differences might have some effect on the outcomes, which differ from that in Western cohorts. Assessing the durability of ART regimens in Asia is imperative if we are to plan accurately for long term ART procurement needs. Understanding the pattern of antiretroviral treatment in different regions of the world to tailor adequate second line and salvage treatment strategies is thus warranted.

The aim of this study is to explore the rates and predictors of the change of combination antiretroviral therapy in clinical practice of treatment naïve patients in the TREAT Asia HIV Observation Database (TAHOD) with a specific emphasis to differences in drug availability across the region.

## Methods

Data from TAHOD, the Therapeutics Research, Education, and AIDS training in Asia (TREAT Asia) HIV observational database, were used in this study. TREAT Asia is a cooperative network of clinicians throughout Asia and the Pacific that aims to expand the capacity for broader introduction of HIV/AIDS in the region. TAHOD is the first collaborative study by the TREAT Asia network. TAHOD involves 15 clinical sites in the Asia and the Pacific region. Criteria for site selection were based on the ability to contribute data in an appropriate format within the initial 3-year period. We also tried to retain sites so as to represent countries across the region. Available funding limited patient recruitment to 200 patients per site. With limited resources, it was thought that recruiting an entirely representative sample of all patients attending a site was unachievable. Instead, the emphasis was placed on recruiting patients who were thought likely to remain in follow-up. Each site identified patients with regular clinic follow-up and then recruited a consecutive sample of such patients, aiming to recruit patients receiving and not receiving antiretroviral treatment at the time of recruitment. Although this recruitment approach does not provide patient samples that are entirely representative of patients attending a site, the expected good follow-up rates ensure that robust analyses can be made regarding the natural history of HIV disease on and off antiretroviral treatment. Ethics approved was obtained from the University of New South Wales and a local committee for each site. Since data were entirely observational, informed consent was not obtained, unless specifically requested by sites local ethics committee. More detail of TAHOD methods is described elsewhere [6,13].

Data collected in TAHOD included 1) demographic data, 2) stage of disease (CD4 and CD8 cell count, HIV-RNA test date and result, AIDS-defining illness [defined according to 1993 Center for Disease Control and Prevention (CDC) revision of the AIDS case definition], and date and cause of death); and 3) treatment. All data were entirely observational, with tests or interventions performed according to clinical guidelines at each clinical site. Data were combined via standardized formats in Microsoft Excel and transferred electronically (compressed with password-protection) to the National Centre in HIV Epidemiology and Clinical Research (NCHECR) for central aggregation and analysis. Ethical approval for the study was obtained from the University of New South Wales Ethics committee and from local Ethics committees.

TAHOD patients commencing their first ART with 3 or more antiretroviral drugs and who had baseline and at least 1 follow-up visit were included in this study. Retrospective and prospective data with follow-up until September 2005 were included in this analysis.

Combination treatment change was defined as any change in ART excluding dosage change. Any start or stop of an individual antiretroviral drug was considered to be a treatment change. An interruption of a drug of less than 14 days was not considered to be a treatment change. Patients who died were assumed to stop treatment on that day, and this was counted as a treatment change.

Country income category was classified according to the World Bank criterion for classifying economies. Four groups divided by 2005 gross national income (GNI) per capita are identified: low income country ($875 or less), lower-middle income ($876 – $3,465), upper middle income ($3,466 – $10,725) and high income country ($10,726 or more) [14].

Availability of antiretroviral treatment at each site was expressed as the number of drugs reported to have ever been used by TAHOD patients seen at that site.

Reasons for stopping treatment are collected in TAHOD by physician report at the time of stopping an individual drug. The physician reports the major reason believed to be underlying the reason for stopping a drug. Reasons include treatment failure, clinical progression/hospitalisation, patient decision/request, compliance difficulties, drug interaction, adverse event and other.

### Statistical analysis

Rate of changing antiretroviral therapy was calculated as the number of events over the person-years follow-up. The time to change for the first, second and third combination was estimated using Kaplan-Meier method. Factors associated with the rate of change were assessed using random-effect Poisson regression methods that allow for multiple treatment changes (events) within individual patients. Factors included were, age at initiation of combination treatment, sex, exposure category (heterosexual, homosexual, IDU and other exposures or unknown), CD4 and viral load at initiation of combination treatment, previous AIDS defining illness, calendar year, number of combination, type of treatment (d4T/3TC/NVP; combinations of ART with NNRTI no PI, other than d4T/3TC/NVP; combinations of ART with PI no NRTI; and NNRTI only), country income category [14] and number of antiretroviral treatment availability in the country. Variables with a p-value less than or equal 0.10 were considered for inclusion in multivariate models. Multivariate models were built using forward stepwise techniques. Variables included in the final multivariate model were assessed for interactions. Overall survival was compared between groups using Cox regression. Time to stopping the first regimen due to toxicities and treatment failures was summarised using a cumulative incidence plot, which allows for the competing risk nature of the data[15,16]. Statistical significance was taken as a 2-sided p-value of less than 0.05. All the analyses were performed using STATA, software, version 8.2 [17]

## Results

### Patient's characteristics

From September 2003 to September 2005, 2979 patients were recruited to TAHOD, including 2345 patients who commenced ART.

Details of patient's characteristic are shown in table 1. The majority of patients were male (71%). The mean age at the first treatment was 36.8 years. The main reported transmission route was heterosexual contact (72%). Distribution of income category among sites participating to TAHOD was well balanced, with 47% of TAHOD participating sites from lower middle income countries, 24% from a low income and 29% from upper middle and high income country according to World Bank report. Twenty six percent of patients were recorded as having a previous AIDS defining illness prior to treatment initiation. 80% of patients were started on an NNRTI based regimen, among whom 37% initiated treatment with a combination of d4T/3TC/NVP. The majority of patients who started with 3 or more drugs including NNRTI, no PI (other than d4T/3TC/NVP) were on 3TC/AZT/EFV (238/791) and 3TC/d4T/EFVI (165/791). The median [range] number of drugs prescribed at least once in each site was 12 [6-16]. The median number [range] of NRTI, NNRTI and PI were 5 [3-6], 2 [2,2] and 5 [1-8], respectively (Table 1).

**Table 1:** Baseline characteristics at first treatment combination (N = 1,846)

**Characteristics**	
**Age**, years mean (SD)	36.9 (10)
**Gender, n (%)**	
- Male	1,324 (71.7)
- Female	520 (28.2)
- Transgender	2 (0.1)
**Ethnicity, n (%)**	
- Chinese	567 (31)
- Indian	375 (20)
- Thai	502 (27)
- Others	402 (22)
**Exposure, n(%)**	
- Heterosexual	1,333 (72)
- IDU +others +unknown	192 (11)
- Homosexual	321 (17)
**Income, n (%)**	
- Low income	451 (24)
- Lower middle income	859 (47)
- Upper middle and high income	536 (29)
**CD4 cells/μL at baseline**, n (%)	
- < 50	432 (23)
- 51 – 100	215 (11)
- 101 – 200	309 (17)
- > 200	232 (13)
- Missing	658 (36)
**HIVRNA at baseline, n (%)**	
- < 100,000	210 (11)
- > 100,000	256 (14)
- Missing	1380 (75)
**Previous AIDS, n(%)**	
- No previous ADI	1363 (74)
- Previous ADI	483 (26)
**First treatment combination, n(%)**	
- d4T/3TC/NVP	676 (37)
- 3 or more ART with NNRTI, no PI (other than d4T/3TC/NVP)	791 (43)
-* 3TC/AZT/EFV*	*238*
-* 3TC/d4T/EFV*	*165*
-* 3TC/AZT/NVP*	*161*
-* ddI/d4T/EFV*	*82*
-* ddI/d4T/NVP*	*68*
-* ddI/AZT/EFV*	*24*
-* ddI/3TC/EFV*	*16*
-* others*	*37*
- 3 or more ART, with PI, no NNRTI	344 (19)
-* Indinavir*	*124*
-* Lopinavir*	*90*
-* Nelfinavir*	*49*
-* Saquinavir*	*33*
-* Atazanavir*	*20*
-* others*	*28*
- 3 or more ART, NRTI only	17 (1)
-* ABC/3TC/AZT*	*8*
-* ddI/3TC/AZT*	*6*
-* others*	*3*
Others	18 (1)
**Median (IQR) number of drugs**	12 (6 – 16)

Individual patient data on ART funding are not collected in TAHOD. However, a site survey showed that all 15 sites that responded to the survey were able to subsidise the first line regimen. Eight sites were able to provide free first line ART to TAHOD patients, while the remaining 7 sites could only partially support the cost of ART for the first line regimen. Only 9 sites out of 15 were able to subsidise a second line regimen, of whom 5 could provide free ART.

### Rate of treatment change

Out of 2345 patients who started ART, 1846 patients started first treatment with 3 or more drugs in combination. The median follow-up of this cohort was 2.4 years. The overall rate of combination antiretroviral treatment change after the first combination treatment was 29 per 100 person-years. Rates for second and third change were 41 per 100 person-years in both changes.

**Figure 1:**
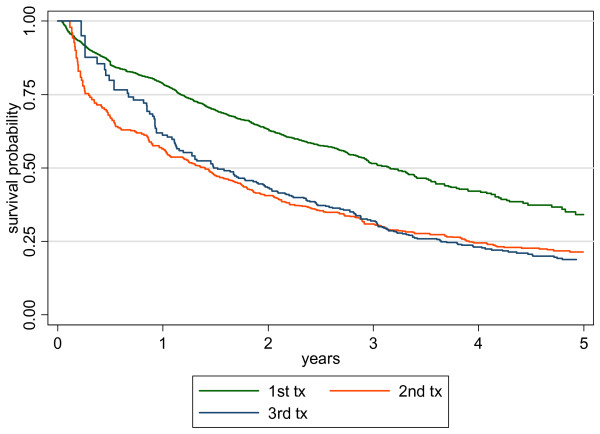
Duration of first, second and third combination treatment.

The median duration of first, second and third treatments are shown in Figure 1. The patients remained on their first combination for a median of 3.2 (1.2 – 6.3) years. Out of the 1846 patients, 719 patients changed their first treatment and 6% of subsequent treatment regimen included a PI. 596 out of 719 (83%) started a second regimen with a median duration of 1.4 (0.3 – 3.9) years. 343 (58%) patients started a third regimen with a median duration of 1.5 (0.7 – 3.6) years.

**Figure 2:**
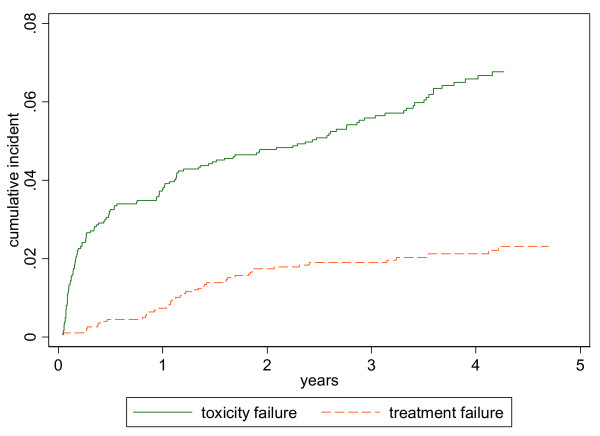
Cumulative incident of toxicity and treatment failure in the first treatment. x axis is "years". Y axis is "cumulative incidence". green line represents toxicity failure. red line represents treatment failure [see Figure 2]

45 patients ceased their first regimen due to treatment failure, with a median treatment duration of 1.2 years. 4, 18 and 19 patients ceased their first regimen due to treatment failure while on d4T/3TC/NVP, other NNRTI based regimen, and PI based regimen respectively, with median durations in these patients of 1.6, 1.2 and 1.2 years respectively. The cumulative incidence of changing the first regimen due to toxicities or treatment failure are shown in Figure 2.

The main reasons reported for stopping first, second and third treatment were adverse events (Table 2). Lipoatrophy was the most common side effect leading to treatment change in the first treatment combination. Anaemia and rash were the most common reason accounting for treatment change in the second and third combinations (Table 3). Toxicities leading to ceasing the first treatment regimen is broken down before and after 6 months in Table 4. Lipoatrophy was more common after 6 months with d4T/3TV/NVP treatment, while rash was more common before 6 months. Anaemia was found only in the first 6 months of treatment (10/129) and none was found in d4T/3TC/NVP regimen.

**Table 2:** Reasons for stopped 1^st^, 2^nd ^and 3^rd ^treatments

	Treatments, n(%)		
Reason	1 (n = 413)	2 (n = 188)	3 (n = 130)
Adverse events	129 (31)	50 (27)	41 (32)
Others	116 (28)	65 (35)	36 (28)
Treatment failure	45 (11)	27 (14)	21 (16)
Patient decision/request	75 (18)	22 (12)	19 (15)
Compliance difficulties	29 (7)	14 (7)	7 (5)
Clinical progression/hospitalisation	11 (3)	9 (5)	1 (0.7)
Drug interaction	8 (2)	1 (0.5)	5 (4)

**Table 3:** Main adverse events for 1^st^**, 2**^nd^**and 3**^rd^

	Treatment, n(%)		
Adverse events	1 (n = 129)	2 (n = 50)	3 (n = 41)
Lipoatrophy	26 (20)	11 (22)	5 (12)
Rash	13 (10)	0 (0)	11 (27)
Anaemia	10 (8)	10 (20)	4 (10)
Neuropathy	5 (4)	4 (8)	1 (2)
Metabolic disturbance	6 (6)	0 (0)	0 (0)

**Table 4:** Toxicities reported as reason for first treatment change before and after 6 months, by treatment (n = 129)

	Treatment (%)					
	
Reasons	d4T/3TC/NVP		with NNRTI, no PI		with PI	
	≤ 6 mths	> 6 mths	≤ 6 mths	> 6 mths	≤ 6 mths	> 6 mths
Others	6 (5)	5 (4)	9 (7)	15	4 (3)	6 (5)
Lipoatrophy	0 (0)	19 (15)	0 (0)	6	0 (0)	1 (0.8)
Rash	7 (5)	1 (0.8)	2 (1.6)	0 (0)	3 (2)	0 (0)
Anemia	0 (0)	0 (0)	7 (5)	0 (0)	2 (1.6)	0 (0)
Hepatitis	5 (4)	2 (1.6)	1 (0.8)	0 (0)	0 (0)	0 (0)
GI symptoms	1 (0.8)	0 (0)	2 (1.6)	0 (0)	4 (3)	0 (0)
Lactic acidosis and hyperlactatemia	0 (0)	3 (2)	0 (0)	2	1 (0.8)	1 (0.8)
Metabolic Disturbance	0 (0)	1 (0.8)	0 (0)	3	0 (0)	1 (0.8)
Peripheral neuropathy	1 (0.8)	0 (0)	1 (0.8)	3	0 (0)	0 (0)

### Combination therapy characteristic at switch

In the participating sites from low income and lower-middle income countries (n = 10/15 sites), 14% of the patients had data available on CD4 T cell count within 3 months before the occurrence of the treatment change. For patients for whom CD4 T cell count was available, the latest CD4 cell count before the switch to the second regimen showed that 9% of patients had CD4 T cell count below 50, 7% had CD4 T cell counts of 51–100 and 21% from 101–200. Similarly in low and lower-middle income countries, HIV-RNA was measured in only 26% of the patients overall before a switch to a second regimen, and in 21% of patients before the switch to third regimen, indicating that these monitoring tests are not routinely performed.

### Predictors of rates of combination treatment change

Table 5 shows factors associated with rates of combination treatment change. In univariate analyses factors related to rate ratios of combination treatment change were; country income category (p = 0.002), drug class combination at baseline (p < 0.001), number of combinations (p < 0.001), calendar year (p = 0.002) and number of drugs available (p = 0.009).

**Table 5:** Factors associated with rates of combination antiretroviral treatment changes

					Univariate^1^	
	N	Follow Up (years)	# events	Rate/follow-up year	RR (95% CI)	p
**Overall**	1846	5121.3	1491	0.29		
**Age**(per 10 years)					1.04 (0.98 – 1.12)	0.220
**Sex**						
Male	1324	3734	1081	0.29	1.00	...
Female	520	1384	408	0.29	1.01 (0.87 – 1.17)	0.905
**Exposure**						0.004
Heterosexual	1333	3770	1052	0.28	1.00	...
IDU + others + unknown	192	433	171	0.40	1.43 (1.16 – 1.78)	0.615
Homosexual	321	918	268	0.29	1.09 (0.91 – 1.30)	0.348
**Income category**						0.004^2^
Low income	451	1079	263	0.24	1.00	...
Lower middle income	859	2319	669	0.29	1.14 (0.95 – 1.36)	0.159
Upper middle high income	536	1723	559	0.35	1.31 (1.09 – 1.58)	0.005
**Previous AIDS**						
No	1363	4569	1331	0.29	1.00	...
Yes	483	552	160	0.29	1.09 (0.94 – 1.27)	0.255
**CD4**						0.317^2^
< 50	432	1147	335	0.29	1.00	...
51 – 100	213	544	145	0.27	0.93 (0.72 – 1.20)	0.549
101 – 200	309	821	224	0.27	0.98 (0.79 – 1.22)	0.884
- > 200	232	725	221	0.30	1.14 (0.90 – 1.28)	0.267
Missing	660	1885	566	0.30		
**HIV-RNA**						
≤ 100,000	210	700	261	0.37	1.00	...
> 100,000	256	752	234	0.31	0.84 (0.66 – 1.07)	0.169
Missing	1380	3669	996	0.27		
**Drug class combination**						<0.001
d4T/3TC/NVP	676	1497	219	0.15	1.00	...
with NNRTI, no PI	791	2392	576	0.24	1.59 (1.35 – 1.88)	<0.001
with PI, no NNRTI	344	926	390	0.42	2.88 (2.40 – 3.45)	<0.001
NRTI only	17	102	72	0.71	5.38 (3.94 – 7.35)	<0.001
Others	18	205	234	1.14		
**Combination**^3^						<0.001^3^
First	1846	3360	764	0.23	1.00	...
Second	719	908	376	0.41	1.22 (1.03 – 1.44)	0.023
Third +	692	853	351	0.41	0.72 (0.58 – 0.89)	0.003
**Calendar year**						
≥ 2003	808	3907	1053	0.27	1.00	...
≤ 1999 – 2002	1038	1214	438	0.36	1.23 (1.10 – 1.40)	0.001
**Number of drugs available**^4^						
> 12	922	2742	739	0.27	1.00	...
≤ 12	924	2379	752	0.32	1.19 (1.04 – 1.36)	0.009

In the multivariate model, the type of regimen used at treatment initiation significantly predicted the rate ratios of subsequent changes (with NNRTI and no PI; RR 1.64 (1.38 – 1.96) p < 0.001, with PI no NNRTI; RR 3.39 (2.76 – 4.16) p < 0.001, NRTI only; RR 6.37. (4.51 – 9.00) p < 0.001, reference regimen is d4T/3TC/NVP). Moreover, being on a second or a third combination regimen was associated with a reduced rate ratio of change in ART, as compared with being on a first prescribed combination therapy (second RR 0.82 (0.68 – 0.99) p = 0.039, third RR 0.77 (0.61 – 0.97), p = 0.024). Sites with fewer than 12 drugs available had an increased rate of treatment changes (1.31 (1.13 – 1.51), p < 0.001). This increased rate of treatment change was largely driven by the large number of drug cessation, with 119 of 752 treatment changes due to simply stopping in sites with fewer than 12 drugs used, compared with 72 of 739 changes in sites with 12 or more drugs used. Exposure category was also found to be associated with rates of treatment changes. In particular, injecting drug users, and other/unknown exposure was found to have an increased rate of treatment change (1.24 (1.00 – 1.54), p = 0.055). Rate ratios from non-statistically significant factors considered for inclusion in multivariate models are also presented in Table 6 adjusted for the statistically significant variables included in the multivariate model. The key variables included in the final multivariate model were also assessed for interaction effects, but no statistically significant interaction effects were found (data not shown).

**Table 6:** Multivariate model for factors associated with rates of combination antiretroviral treatment changes

	Multivariate^5^	
	RR (95% CI)	p
**Drug class combination**		**<0.001**
d4T/3TC/NVP	**1.00**	**...**
with NNRTI, no PI	**1.64 (1.38 – 1.96)**	**<0.001**
with PI, no NNRTI	**3.39 (2.76 – 4.16)**	**<0.001**
NRTI only	**6.37 (4.51 – 9.00)**	**<0.001**
Others		
**Combination**^3^		**0.019**^3^
First	**1.00**	**...**
Second	**0.82 (0.68 – 0.99)**	**0.035**
Third +	**0.77 (0.61 – 0.97)**	**0.024**
**Number of drugs available**^5^		
> 12	**1.00**	**...**
≤ 12	**1.31 (1.13 – 1.51)**	**< 0.001**
**Exposure**		**0.047**
Heterosexual	**1.00**	**...**
IDU + others + unknown	**1.24 (1.00 – 1.54)**	**0.055**
Homosexual	**0.88 (0.73 – 1.07)**	**0.209**
**Income category**		0.824
Low income	1.00	...
Lower middle Income	1.25 (0.98 – 1.56)	0.068
Upper middle high income	1.06 (0.85 – 1.33)	0.581
**Calendar year**		
≥ 2003	1.00	...
≤ 1999 – 2002	1.13 (0.98 – 1.30)	0.086

1 Estimated univariate Relative rates (RR) from random effects Poisson model may not equal ratio of crude rates

2 p from test for trend

3 Second and third combinations included patients who were not on combination treatment

4 Total number of drugs that have been used in TAHOD patients at the site where patients were receiving care

5 Variables included in the final multivariate model are presented in bold. All other non-significant variables are also presented adjusted for the variables included in the final multivariate model.

### Survival by income category

There were 34 deaths in patients included in these analyses, an overall mortality rate of 6.6 per 1,000 person years. Compared to low income countries, survival in high income countries was not statistically significantly raised, (hazard ratio = 1.6, (95% CI; 0.5 – 5.3), p = 0.414).

## Discussion

We analysed the pattern of ART changes in various Asian sites participating in TAHOD. The patients remained on their first combination for a median of 3.2 (1.2 – 11.7) years. The overall rate of combination treatment change in this cohort was 29 per 100-person year. We observed significantly higher treatment duration in sites located in low income countries as compared with sites from higher income countries (p < 0.001).

These differences could have been expected from previous report generated from data in the US and Australia's cohorts. Chen et al showed that the median duration of a first combination did not exceed 1.6 years [8]. Pallela et al in the HIV Outpatient Study (HOPS) in the USA have shown even shorter duration with only 11.8 months spent on the first prescribed regimen[18]. The Australian HIV observational database used the same criteria as ours to define rate of changes and has the same number of years of follow-up (2.3 years) [8,9,18]. The rate of combination antiretroviral treatment change in The Australian HIV Observation Database (AHOD) was 0.45 combinations per year, which is higher than our result of 0.29 combinations per year [9]. There are however substantial differences between the 2 study populations. In TAHOD about 50% of patients started their first treatment with CD4 less than 200 cells/μL compared with 23% in AHOD. We found 26% of TAHOD reported having a previous AIDS defining illness while only 11% reported in AHOD. Thus, our data suggest that despite advanced disease, patients in TAHOD tolerate well the first prescribed regimen and change at a much slower rate than in AHOD.

We also found that the rate of treatment change in the second and third regimens were at a slower rate than the first treatment (relative rates of 0.81 and 0.70 respectively). This contrasts with results from AHOD which found that the rate of change did not change statistically significantly in second and third combinations[9]. This may also be a reflection on how treatment availability impact on the treatment strategies. Even though the results from both Western cohorts showed shorter time on first treatment, it should be noted that these findings were based on data from an earlier period when there were fewer antiretroviral treatments available, a greater proportion of patients previously treated with mono and double therapy, and arguably that physicians were less experienced, factors that could affect rates of treatment change.

Our results tend to illustrate that in the context of limited resources, where the first regimen appears to be by far the cheapest option, clinicians might be reluctant to switch even in the context of true virological failure to alternative more expensive options if the patient is not clinically symptomatic, thereby running the risk that they jeopardize the chances of finding a successful regimen later on. This risk, however, is only present if the reason for switching is virological failure. It is also the case that HIV viral load testing is often not routinely performed in low income countries, meaning that true virological failure may not be detected. Alternatively, if second or third line regimens are relatively unaffordable or not available, this may influence decisions to even perform HIV viral load testing. This could to some extent explain the low rate of treatment changes in TAHOD as compared to AHOD, despite patients at more severe disease stages. Indeed, when looking for predictors of treatment changes, income category was significantly associated only in the univariate model. Beside the effect of different treatment options being different from country to country, we also analysed the effect of the type of first prescribed regimen in our study. Patients who started with d4T/3TC/NVP stopped or changed their treatments at a slower rate than patients who started their treatment with other regimens. This confirms previously published report from TAHOD on a smaller sample of patient (n = 404) [11]. This also could be linked to the fact that this combination is the cheapest available. It could also be related to TAHOD being relatively young and the follow-up time still relatively short, so that long-term, chronic d4T related mitochondrial toxicity will be seen more frequently with longer follow-up. Further results from TAHOD have shown that patients who were on a d4T based regimen were more likely to cease treatment than patients who were on an AZT based regimen after greater than 9 months treatment [19].

1,846 patients started an NNRTI based regimen as first combination. Of the 719 who stopped or started any drug, 6% switched to a PI based regimen. Moreover, only a minority of the switches have been triggered by the usual surrogate markers used in Western countries, such as CD4 and VL. As already shown in TAHOD [11], this analysis confirms the hypothesis that most of the switches are due to toxicity or tolerance issues rather than related to treatment failure at least in the short or medium term. It may also be that clinicians are reluctant to put the patients on a second regimen when limited treatment options are available. The low rates of treatment changes on second and third combinations may reflect scarcity of affordable or available salvage options rather than durability of regimens. Even though income category was not statistically significant in the multivariate models, it is worth noting that rates of changing treatment were slower in low income countries in univariate models, and this became non-significant on adjustment for type of ART regimen. This seemingly high threshold to switch therapy among clinically stable patients probably reflects persisting with cheaper, generic treatment regimens in patients who are either failing virologically or do not have HIV viral load tests available, and raises issue regarding development of drug resistance. TAHOD plans to address these issues in future analyses based on studies of drug resistance. We were unable to separate individual patients who received free ART from those who had to pay for their own treatments. We had, however, site details about free access to ART and the majority of patients were able to access to free ART. This might not be the real practice in Asian countries as these sites are mainly academic sites, and patients might also participate in clinical trials.

There were some limitations in this study. First, we used retrospective and prospective data. This limitation led to some gaps in information about why patients stopped their treatment which might be clinically relevant. Based on prospective data, we found that 31% of patients stopped their treatment because of adverse events in the first treatment, 27% and 32% in the second and third treatment combination, respectively. Lipoatrophy is the major reason for patients to stop their treatment. Second, TAHOD patients might not be completely representative of HIV-infected patients in Asia-pacific region: only patients with a good follow-up (according to the physician's opinion) are recruited. Furthermore TAHOD sites are generally located at academic centres in the region. Care should be taken in extrapolating our results to all patients treated in the Asia-Pacific region. Third, because individual patient data were not available, the country income category was measured at an ecological level using the World Bank classification. This might not truly represent individual patient's income in some sites. In particular, it is likely that patients from a site in a nominal low income country are of higher income status than is typical, or that HIV patients from a nominal high income country are from a lower income status within that country. It may be that patients seen at nominal low income sites have a greater range of treatment options than would be typical, especially since our sites are mainly from academic centres. Similarly, the number of drugs available is an ecological variable based on the total number of drugs that had been used in TAHOD patients at a given site. Not all these drugs may be available to all TAHOD patients, and so may overestimate drug availability. Fourth, we collect the main reason as reported by the physician for stopping treatment, but reasons for stopping are often interrelated. For example, clinical progression and treatment failure are often related. Patient request may reflect financial difficulty or toxicity. Reasons for stopping treatment cannot be further delineated in TAHOD, and should be interpreted cautiously.

Our study found lower rates of antiretroviral treatment change than in developed country cohorts. Within TAHOD, higher income countries had a greater rate of antiretroviral treatment change than low income countries, although this difference disappeared on adjustment for other treatment variables. A lower total number of drugs available was also associated with a greater rate of treatment change, and in particular more treatment cessations rather than switches. Taken together, this suggests that drug availability does impact the strategies used by clinicians to change the antiretroviral regimen. A recent report from the Global Fund has shown that any increase of alternative first line and second line drugs will more than double the budget allocated to ART drugs in some programs. Forecasting the need in terms of treatment regimen in a region with a high patient load is therefore a key issue. Resources should be made available for patients to have access to a wider range of treatment options.
